# Comparison of the fracture toughness and wear resistance of indirect composites cured by conventional post curing methods and electron beam irradiation

**DOI:** 10.4103/0972-0707.71647

**Published:** 2010

**Authors:** C Vaishnavi, S Kavitha, L Lakshmi Narayanan

**Affiliations:** Department of Conservative Dentistry & Endodontics, SRM Kattankulathur Dental College, Tamil Nadu, India

**Keywords:** Electron beam irradiation, fracture toughness and wear resistance, indirect composites, post curing

## Abstract

**Aim:**

To compare the fracture toughness and wear resistance of indirect composites cured by conventional post curing methods and electron beam irradiation.

**Materials and Methods:**

Forty specimens were randomly assigned into four groups of ten each and were subjected to various post curing methods. Fracture toughness and wear resistance tests were performed and the results were tabulated and analyzed statistically using Kruskal Wallis and Mann-Whitney U test.

**Results:**

It was found that Inlay system showed higher values followed by electron beam irradiation.

**Conclusion:**

Electron beam irradiation of dental composites gives comparable mechanical properties to other post curing systems. It can be concluded that further studies with increased radiation dose should be performed to improve the mechanical properties of indirect composites.

## INTRODUCTION

The ever rising demand for aesthetics has led to the wide clinical acceptance of composite resin restorations. However, direct restorations have their own inherent limitations like incomplete polymerization and poor mechanical properties. Studies show that indirectly fabricated composites overcome these limitations.

The various advantages of indirect composites include better polymerization, better mechanical properties, better contacts and contours. The enhanced mechanical properties may largely be because of increased degree of conversion, which leads to increase in cross-linking. An increase in mechanical strength of the restoration, especially resistance to fracture and wear are the most important requisites for posterior restorations. Several post curing methods have been advocated to increase the polymerization, like curing in Nitrogen atmosphere, curing under heat and pressure or curing in inlay systems designed by the manufacturer.[1] One of the latest approaches in this direction is electron beam irradiation, which has been claimed to change the mechanical properties of the polymer.

Therefore, this study was done to evaluate the fracture toughness and wear resistance of indirect composites that were subjected to electron beam irradiation as a post curing method and compare with conventional methods which included autoclave and the inlay system.

## MATERIALS AND METHODS

The material used in this study was Adoro (D3, IvoclarVivadent, Liechtenstein), an indirect composite resin.

### Study I: Evaluation of fracture toughness

Forty specimens were prepared using split steel mould with the dimensions of 14 mm × 2mm × 2mm (length × width × height). The composite material was placed in the steel mould and light pressure was applied with the glass slide to remove the excess. Light curing was done for 20 sec using Lumamat light curing device. The specimens were then randomly assigned into four groups of ten each.

### Post curing protocol

Group I - The specimens were subjected to electron beam irradiation using an electron beam accelerator (Siemens) at a dose of 1 KGy and 6 MeV.

Group II - The specimens were cured under Lumamat 100 inlay system (specifically designed for Adoro material by the manufacturer) for 25 min. The temperature used in this system was 104°C.

Group III - The specimens were cured under heat and pressure (autoclave) at 121°C for 10 min.

Group IV - The specimens were not subjected to any post curing methods (control group).

A 0.5 mm wide notch was created at the midspan of the 14 mm long specimen and was subjected to three point bending test with a constant cross-head speed of 1mm/min using Universal Testing machine (LR 100K, Lloyd Instruments, USA). The fracture toughness (K1C) was calculated from the formula of fracture mechanics

$${\mathrm{KIC}}_{\left(\max\right)}\;=\;\frac{P_{\max}\;\times \;S}{B\;\times \;H^{3/2}}\;f\left(X\right)$$

$$f\left(x\right)\;=\;\frac{3x^{1/2}\;\left\{1.99-\times \left(1-\times \right)\left(2.15-3.93\times 2.7\mathrm{x2}\right)\right\}\;}{2\left(1+2X\right){\left(1-X\right)}^{3/28}}$$

X = a/H

Where,

S – support distance (14 mm), B – width of the specimen (2 mm), H – height of the specimen (2 mm), a – notch length (0.5 mm), P – fracture load

### Study II: Evaluation of wear resistance

**Figure 1:**
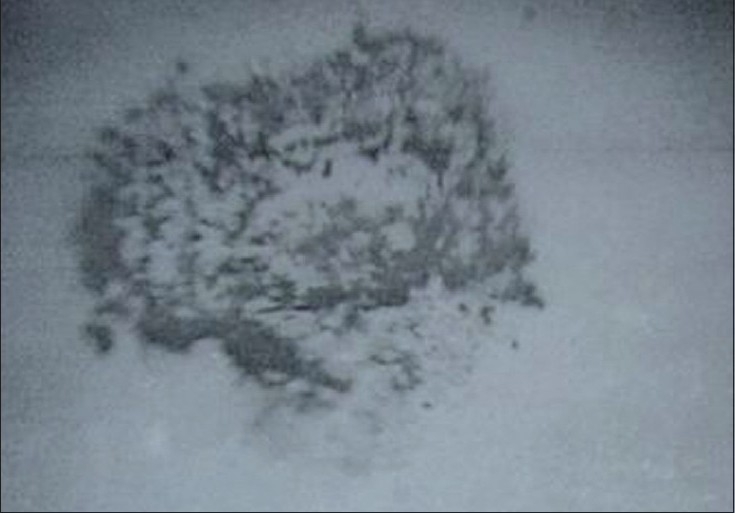
Wear scar diameter

Forty specimens with dimensions of 6 mm × 2 mm (diameter × height) were prepared using brass rings. The excess material was removed with the help of cellophane strips. Light curing was done for 20 sec using Lumamat light curing device. The specimens were randomly assigned into four groups of ten each as described in study I. After initial light curing, the specimens were subjected to different post curing methods as described in study I. Wear tests were performed using a wear test device, under the load of 2kg. Flat specimens were slid against a polished stainless steel ball of 10 mm diameter. Reciprocating motions with an amplitude of 200*µ* were employed and the frequency used was 5 Hz for 2 h. The total number of cycles tested was 36000. The wear test was conducted at room temperature 29°C / 50% RH. Wear loss was quantified based on the wear scar diameter, measured using an optical microscope and image analyzer. The wear scar diameter was measured for Group I, Group II, Group III and Group IV [Figure 1] and the wear volume loss was calculated using wear volume determination formula.[2]

$$V_{1}\;=\;\frac{{\mathrm{\pi h}}_{\mathrm{cap}}^{2}\left(3\mathrm{R-}h_{\mathrm{cap}}\right)}{3}$$

$$R_{\mathrm{fc}}\;=\;\sqrt{\frac{d_{1}d_{\mathrm{II}}}{4}}$$

$$d_{\mathrm{II}}\;=\;\frac{d_{1\;\mathrm{flat}}\;+\;d_{\mathrm{II}\;\mathrm{flat}}}{2}$$

$$d_{I}\;=\;\frac{d_{1\;\mathrm{flat}}\;+\;d_{\mathrm{II}\;\mathrm{flat}}}{2}$$

## RESULTS

The results obtained for both the tests were analyzed using Kruskal Wallis’ one way ANOVA and Mann-Whitney U test. The mean and standard deviation values for fracture toughness test are shown in Table 1. The highest mean value was seen in Group II (the inlay system) followed by Group I (Electron Beam Irradiation), Group III (autoclave) and the lowest value was seen in Group IV (control). The results obtained were statistically significant (*P*<0.05).

**Table 1:** Mean and standard deviation of fracture toughness values (MPa)

Group	Mean ± S.D.	*P* value	Significance at 5% level
I	0.102 ± 0.002	< 0.05	I Vs II, III, IV
II	0.112 ± 0.002	< 0.05	II Vs I, III, IV
III	0.080 ± 0.001	< 0.05	III Vs I, II, IV
IV	0.064 ± 0.002	< 0.05	IV Vs I, II, III

The mean and standard deviation values for wear test are shown in Table 2. The highest mean value was seen in Group IV (control) followed by Group III (autoclave), Group I (Electron Beam Irradiation) and the lowest was in Group II (Inlay system). The results obtained were statistically significant (*P*<0.05).

**Table 2:** Mean and standard deviation of wear scar diameters values (mm)

Group	Mean ± S.D.	*P* value	Significance at 5% level
I	0.805 ± 0.004	< 0.05	I Vs II, III, IV
II	0.761 ± 0.003	< 0.05	II Vs I, III, IV
III	0.968 ± 0.004	< 0.05	III Vs I, II, IV
IV	1.114 ± 0.070	< 0.05	IV Vs I, II, III

## DISCUSSION

The ability to withstand loads in the occlusal areas is a prime requisite for any restoration.[3,4] Over the past, several methods have been evolved to optimize the properties of indirect dental composites. The commonly used methods include curing in nitrogen atmosphere, autoclave, Inlay system etc. Studies have shown that electron beam irradiation can enhance the properties of composites. This study was performed to determine the effects of post curing methods on indirect restorations and hence an indirect composite material Adoro (Ivoclar Vivadent) was chosen for the study. There are no studies which compare electron beam irradiation with other conventional methods. Hence this comparative evaluation was taken up for the study.

Electron beam irradiation is shown to improve the mechanical properties of polymer. It has been widely used in the industrial arena to improve the properties of polymers such as polyethylene, polycarbonate and polysulfone.[5] The two main reactions that occur when a polymer is subjected to electron beam irradiation are chain breakage and chain linkage.[5,6] When breakage of chains occurs at the region of entanglement, there is induction of dense packing. This is probably because of alterations in the molecular arrangement.[7] The bond between the filler and the matrix is also significantly increased.

The degree of cross linking that is produced in the polymer is proportional to the radiation dose.[6,8] Some studies have utilized a radiation dose as high as 200 KGy.[9] But in this study, the lowest possible dose was used to determine whether it could bring about a perceptible enhancement of properties. Hence, a dosage of 1 KGy was used in this study.

Fracture toughness refers to the maximum stress that the material can withstand before fracture occurs.[10] The fracture toughness was evaluated using a Universal Testing Machine. Although these machines do not reproduce the exact clinical scenario, it is a less technique sensitive procedure, giving quantitative results that can be correlated clinically and thus was chosen for the study. Wear resistance was done to determine the resistance of composite resins to abrasion. Two body abrasion test was used because it is a commonly used method to rank the wear resistance of restorative material.[1] Wear resistance largely depends on the filler particles (glass or quartz) and on the silanation of the filler. So wear resistance is improved when there is dense packing between the matrix and the filler. In this study, wear tests were performed using a wear test device and the material loss was measured using an optical microscope. Wear scar diameter is inversely related to wear resistance.

Fracture toughness and wear resistance were higher in Group II (Inlay system) followed by Group I (Electron Beam Irradiation), Group III (autoclave) and Group IV (control). The values were higher in Group II when compared to other groups which could probably be because of the Lumamat 100 Inlay system which is specifically designed by the manufacturer for Adoro material with desired time, temperature and intensity. Group I (Electron Beam Irradiation) showed higher values than other two groups (Group III and GroupIV), but lower values than Group II. This could probably be because of minimal amount of radiation dose (1KGy) chosen for this study. Use of additional amount of radiation could have improved the mechanical properties further.

Although autoclaving gave lower values in comparison to Group I and Group II, its values were higher when compared to the controls. This could be because of the presence of both heat and pressure compared to controls which had only light as the source.

## CONCLUSION

As per the limitations of this study, it can be concluded that electron beam irradiation of dental composites gives comparable mechanical properties to other post curing systems. Increasing the radiation dose would further optimize the material properties. Further studies have to be done with increased radiation dosage before a final conclusion is drawn.
